# Retraction: Epigenetic Silencing of Peroxisome Proliferator-Activated Receptor γ Is a Biomarker for Colorectal Cancer Progression and Adverse Patients’ Outcome

**DOI:** 10.1371/journal.pone.0227376

**Published:** 2019-12-27

**Authors:** The PLOS ONE Editors

Following publication of [1], concerns were raised about results reported in Figs 1, 2, and 5:

- Fig 1E M4-T-M panel lanes 4–8 and C+ appear similar to M1-NM-U panel lanes 1–6 and to Fig 2B M3-U panel lanes 1–6;
- Fig 1E M1-NM-U panel appears similar to Fig 2B M3-U panel;
- In Fig 5F, the Control panel appears similar to the sh-CC panel for the invasion assay. The authors commented that this resulted from an error in figure preparation.

The authors provided original data, where available, as well as replication experiments to support the results in the article but explained that the data underlying the panels of concern are no longer available. The submission of replication data, however, does not address the concerns raised about the similarities in the figures identified above, and we have been unable to resolve these concerns due to the unavailability of the original image data.

As of the date of this retraction, no concerns have been raised to *PLOS ONE* about other figures in the article.

The authors noted that primary data underlying Fig 1A β-actin panel lanes 1–8, Fig 1C, Fig 1G, Fig 2A, 2B and 2D, Fig 4A, 4D and 4F and Fig 5D, 5F, 5G and 5I are unavailable. Original data supporting the other figures, as well as replication data supporting several results, are available from the authors.

The authors apologize for any inconvenience this retraction may cause for readers and regret not being able to provide the underlying data for the panels of concern because, according to the authors, the data was lost during the time passed. The authors furthermore claim that the results reported in the paper are robust and the conclusions are valid.

However, in light of the unresolved concerns about the results reported in Figs 1, 2 and 5, the *PLOS ONE* Editors retract this article.

LS, LA, and V Colantuoni did not agree with the retraction. MP, A Fucci, CA, agree with the retraction. Per the corresponding author, NF, A Febbraro, DP, NN, also agree with the retraction. V Carafa and AN did not clarify directly to the journal whether they agree or disagree with the retraction. V Carafa, AN, and LA noted that they stand for the results in Fig 3 for which they provided the original data corresponding to those published. The original author contributions for the article state: “Conceived and designed the experiments: MP LA VC. Performed the experiments: MP LS A. Fucci VC AN CA NN. Analyzed the data: MP LS A. Fucci VC AN LA VC. Contributed reagents/materials/analysis tools: LS A. Fucci AN NF A. Febbraro DP CA NN. Wrote the paper: MP LA VC.”
